# Antibody-mediated inhibition of HIV-1 elicited by HIV-I DNA priming and boosting with heterologous HIV-1 recombinant MVA in healthy Tanzanian adults

**DOI:** 10.1186/1742-4690-9-S2-O53

**Published:** 2012-09-13

**Authors:** A Joachim, C Nilsson, S Aboud, EF Lyamuya, M Robb, M Marovich, C Ochsenbauer, B Wahren, E Sandström, G Biberfeld, G Ferrari, VR Polonis

**Affiliations:** 1 Muhimbili University of Health and Allied Sciences, Dar es Salaam, Tanzania, United Republic of; 2 Karolinska Institutet / Institute for Infectious Disease Control (SMI), Stockholm, Sweden; 3 Muhimbili University of Health and Allied Sciences (MUHAS), Dar es Salaam, Tanzania, United Republic of; 4 The Henry M. Jackson Foundation, Rockville, MD, USA; 5 Walter Reed Army Institute of Research (WRAIR), Rockville, MD, USA; 6 Department of Medicine, University of Alabama at Birmingham, Birmingham, AL, USA; 7 Venhälsan, Karolinska Institutet (KI) at Södersjukhuset, Stockholm, Sweden; 8 Department of Surgery, Duke University Medical Center, Durham, NC, USA

## Background

We evaluated HIV antibody (Ab) responses elicited by immunization, in a phase I/II placebo-controlled double blind trial using multiclade, multigene HIV-1-DNA prime boosted with HIV-MVA conducted among healthy volunteers in Tanzania (HIVIS03).

## Methods

Sixty HIV-uninfected volunteers, randomized into groups of 20 received placebo or 1 mg HIV-DNA intradermally (id) or 3.8 mg intramuscularly (im). DNA plasmids containing HIV-1 gp160 subtypes A, B, C; rev B; p17/p24 gag A, B and RTmut B were given at months 0, 1 and 3 using a needle-free Biojector device. HIV-MVA expressing CRF01_AE HIV-1 env, gag, pol was administered im by needle at months 9 and 21. Sera were tested at baseline, two months post-first and four weeks post-second HIV-MVA boosting. HIV Ab responses were tested using pseudoviruses and TZM-bl cells as well as luciferase-expressing infectious molecular clones (IMC-LucR) in PBMC-based assays. ADCC responses were tested using the flow cytometry GranToxiLux-based assay.

## Results

Neutralizing Ab activity was demonstrated only in the PBMC assay, and after the second MVA boost in 24 (83%) of 29 vaccinees against the clade CRF01_AE CM235 IMC and in 21 (72%) of 29 vaccinees against clade B SF162-IMC. NK cell depletion from PBMC targets resulted in a significant loss of HIV inhibition by vaccinee sera, indicating a role of Ab-mediated Fcγ-receptor function. Vaccine-induced ADCC responses were detected in 21 (75%) of 28 vaccinees after the second HIV-MVA boost. ADCC Ab titers did not differ significantly between id- (median 840, range 300-5400) and im-primed (median 880, range 400-3600) vaccinees (p=0.45).

## Conclusion

HIV-DNA priming followed by two HIV-MVA boosts elicited HIV-specific inhibitory and/or ADCC-mediating antibody responses in a high proportion of Tanzanian adults.

